# Supplementary material for: Fibrogranular materials function as organizers to ensure the fidelity of multiciliary assembly
Source: Nat Commun. 2021 Feb 24;12:1273. doi: 10.1038/s41467-021-21506-8 (PMC7904937; doi:10.1038/s41467-021-21506-8)
Supplement: Supplementary file 1 — Supplementary Figures [file 41467_2021_21506_MOESM1_ESM.pdf]

Supplementary Information

Fibrogranular materials function as organizers to ensure the fidelity of multiciliary assembly

Huijie Zhao, Qingxia Chen, Fan Li, Lihong Cui, Lele Xie, Qiongping Huang, Xin Liang, Jun Zhou, Xiumin Yan & Xueliang Zhu

State Key Laboratory of Cell Biology, CAS Center for Excellence in Molecular Cell Science, Shanghai Institute of Biochemistry and Cell Biology, Chinese Academy of Sciences; University of Chinese Academy of Sciences, China

Running title: Fibrogranular materials control multicilia fidelity

* Corresponding authors (xlzhu@sibcb.ac.cn and yanx@sibcb.ac.cn)

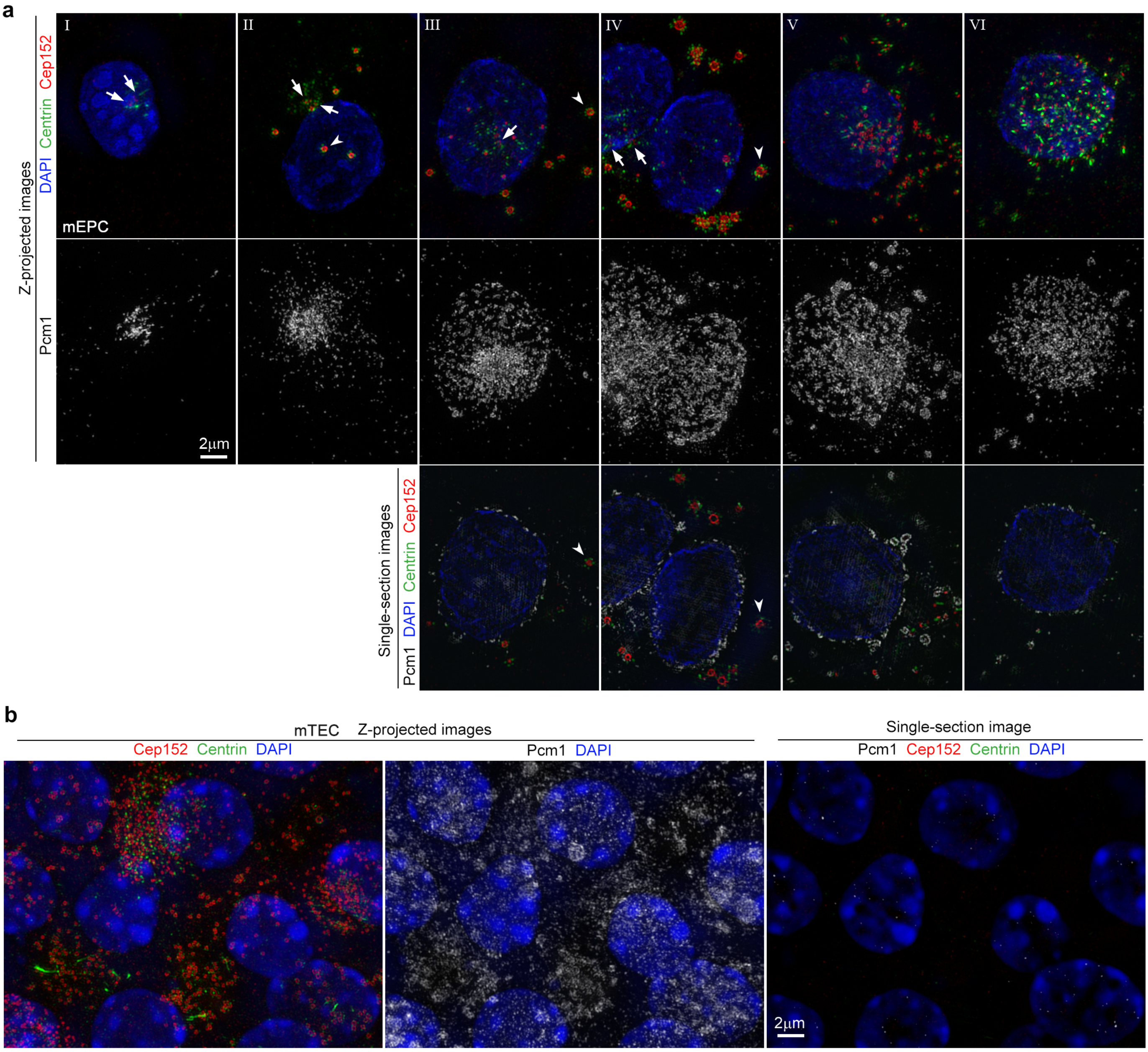

Supplementary Figure 1. Pcm1 decorates both cytoplasmic and perinuclear FGMs in mEPCs (related to Figs 1,2). Cep152 and Centrin respectively served as deuterosome and procentriole markers. The nucleus was visualized by DAPI staining. **(a)** mEPCs at day 3 post serum starvation were immunostained and imaged by 3D-SIM. Arrows and arrowheads indicate parental centrioles and typical deuterosomes, respectively. The single optical sections across the nuclear equators show perinuclear localization of Pcm1. The stages (I-VI), defined previously[26], are similar to those for mTECs[8], except that basal bodies in stage-VI mEPCs are not grouped into multiple clusters (please refer to Figure 1b for a comparison). This makes it sometimes difficult to clearly discriminate mEPCs in stages V and VI. Note that the enrichment of Pcm1 around the parental centrioles starts from stage I, whereas its nuclear surface localization becomes prominent from stage III. **(b)** Pcm1-decorated FGMs did not display nuclear association in mTECs. mTECs were fixed at day 3 after culturing at an air-liquid interface (ALI) and imaged by 3D-SIM.

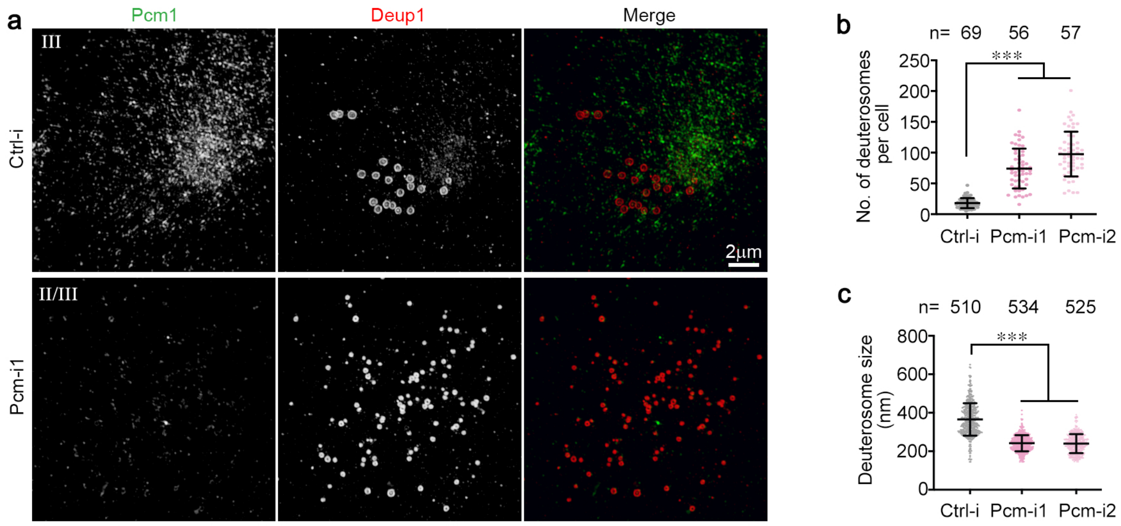

Supplementary Figure 2. Depletion of Pcm1 alters deuterosome number and size (related to Fig. 2c-e). **(a)** 3D-SIM images of representative mEPCs treated as in Figure 2a. Deup1 served as deuterosome marker. **(b,c)** Quantification results (mean ± s.d. with sample dots) of deuterosome number **(b)** and size **(c)**, measured with 3D-SIM images as in **(a)** from three independent experiments. Two-tailed Mann-Whitney *U*-test: *** P<0.001. Source data are provided in the Source data file.

Supplementary Figure 3, Zhao et al.

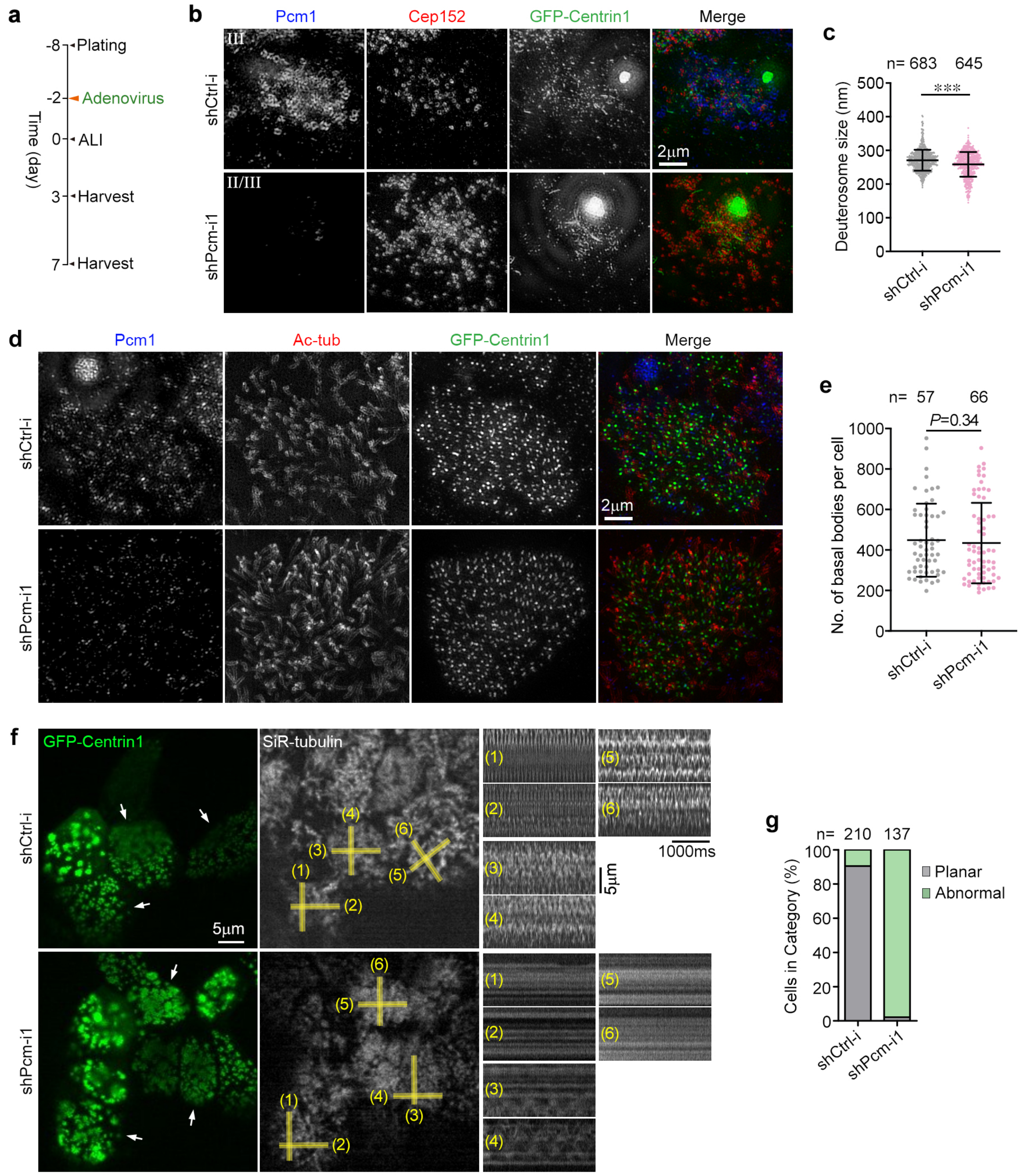

Supplementary Figure 3. Depletion of Pcm1 in mTECs causes increased deuterosome formation and abnormal ciliary motility (related to Figs 2,3).

Quantification results were pooled from two independent experiments. Mean ± s.d. and sample dots are presented in **(c,e)**. Two-tailed Mann-Whitney *U*-test: *** P<0.001. **(a)** Experimental scheme. mTECs were infected with adenoviral particles to co-express shRNAs (shCtrl-i and shPcm-i1) and GFP-Centrin1, which served as both infection and centriole markers. The cells were harvested at day 3 for experiments in **(b)** or day 7 for experiments in **(d,f)**. **(b,c)** Depletion of Pcm1 increased deuterosome number **(b)** and decreased deuterosome size **(c)**. In the 3D-SIM images **(b)**, Cep152 served as deuterosome marker. Note that deuterosomes in the Pcm1-depleted mTECs frequently overlapped or clustered **(b)** to preclude convincing quantification of the numbers. Deuterosome sizes in at least 10 cells were measured in each experiment and condition **(c)**. **(d,e)** Depletion of Pcm1 did not affect multiciliogenesis. In the 3D-SIM images **(d)**, Ac-tub served as cilia marker. Basal bodies in at least 20 cells were quantified in each experiment and condition **(e)**. **(f,g)** Depletion of Pcm1 altered ciliary motility. Multicilia in living mTECs were fluorescently labeled with SiR-tubulin and imaged at 15-ms intervals for 3 sec. The micrographs **(f)** were cropped from the first and second frames of Supplementary Movie 4. Kymographs at two orthogonal positions were generated for the time-lapse images in each of the representative shRNA-expressing (GFP-Centrin1-positive) cells (arrows). At least 54 GFP-Centrin1-positive MCCs were scored for the ciliary beat patterns **(g)** in each experiment and condition. Source data are provided in the Source data file.

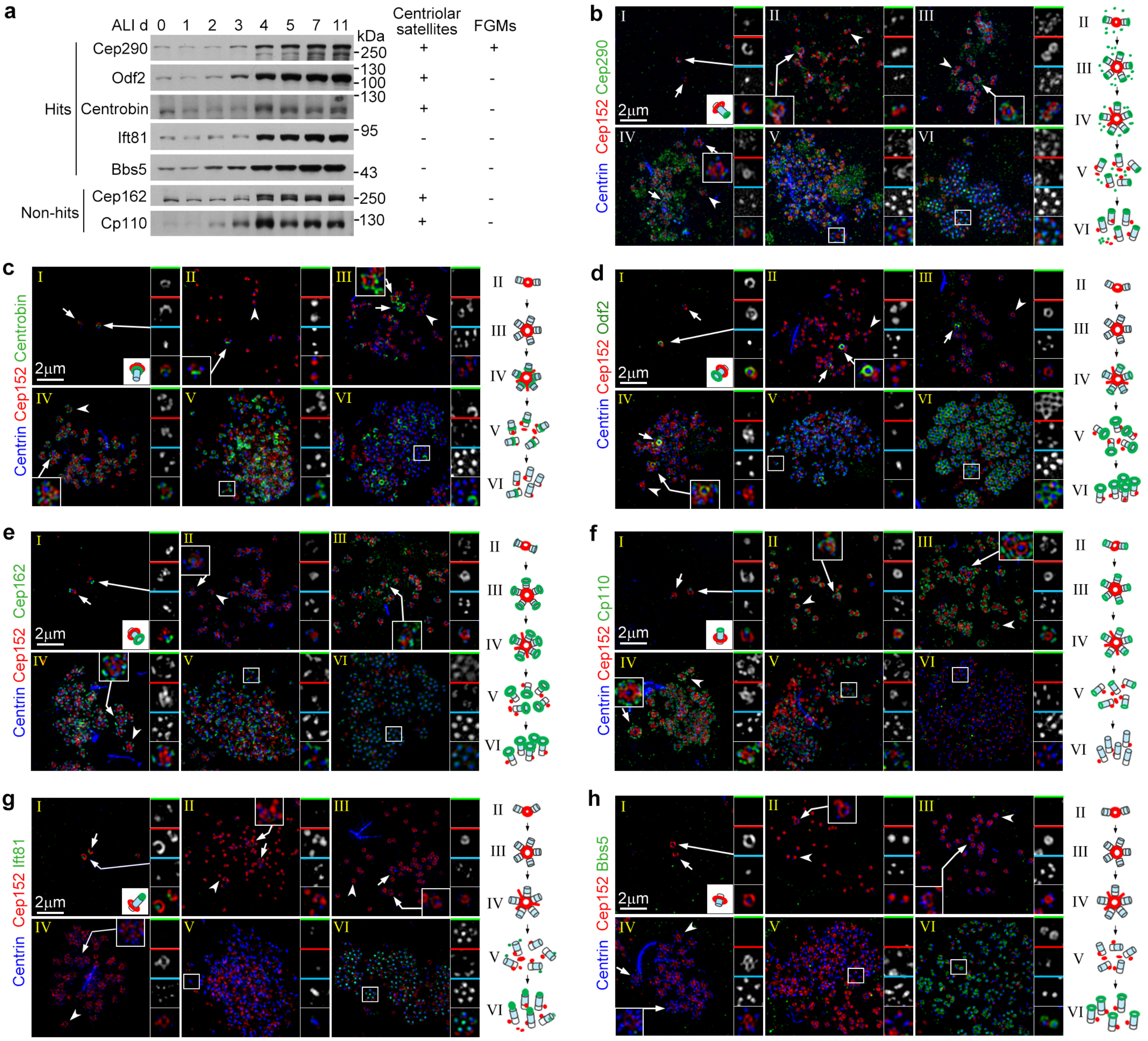

Supplementary Figure 4. Subcellular localizations of several centriolar or ciliary proteins during the centriole amplification of mTECs (related to Figs 4,5).

(a) Expression patterns and properties of the indicated proteins. mTEC lysates and loadings were the same as those in Figure 4f. Hits: proteins emerged in Supplementary Table 1. Non-hits: proteins that were not hit in our differential mass spectrometric analysis. Components of centriolar satellites 15 and/or FGMs (based on **b-h**) are indicated. Representative images from two independent repeats are presented. **(b-h)** Representative 3D-SIM images of mTECs at day 3 for the proteins in **(a)**. Arrows indicate parental centrioles, one of which was magnified. Arrowheads and frames indicate typical regions containing deuterosomes and nascent centrioles, respectively, that were magnified to show details. The diagrams illustrate the protein localizations in stage-I parental centrioles and during the deuterosome-mediated centriole biogenesis (II-VI). Source data are provided in the Source data file.

Supplementary Figure 5, Zhao et al.

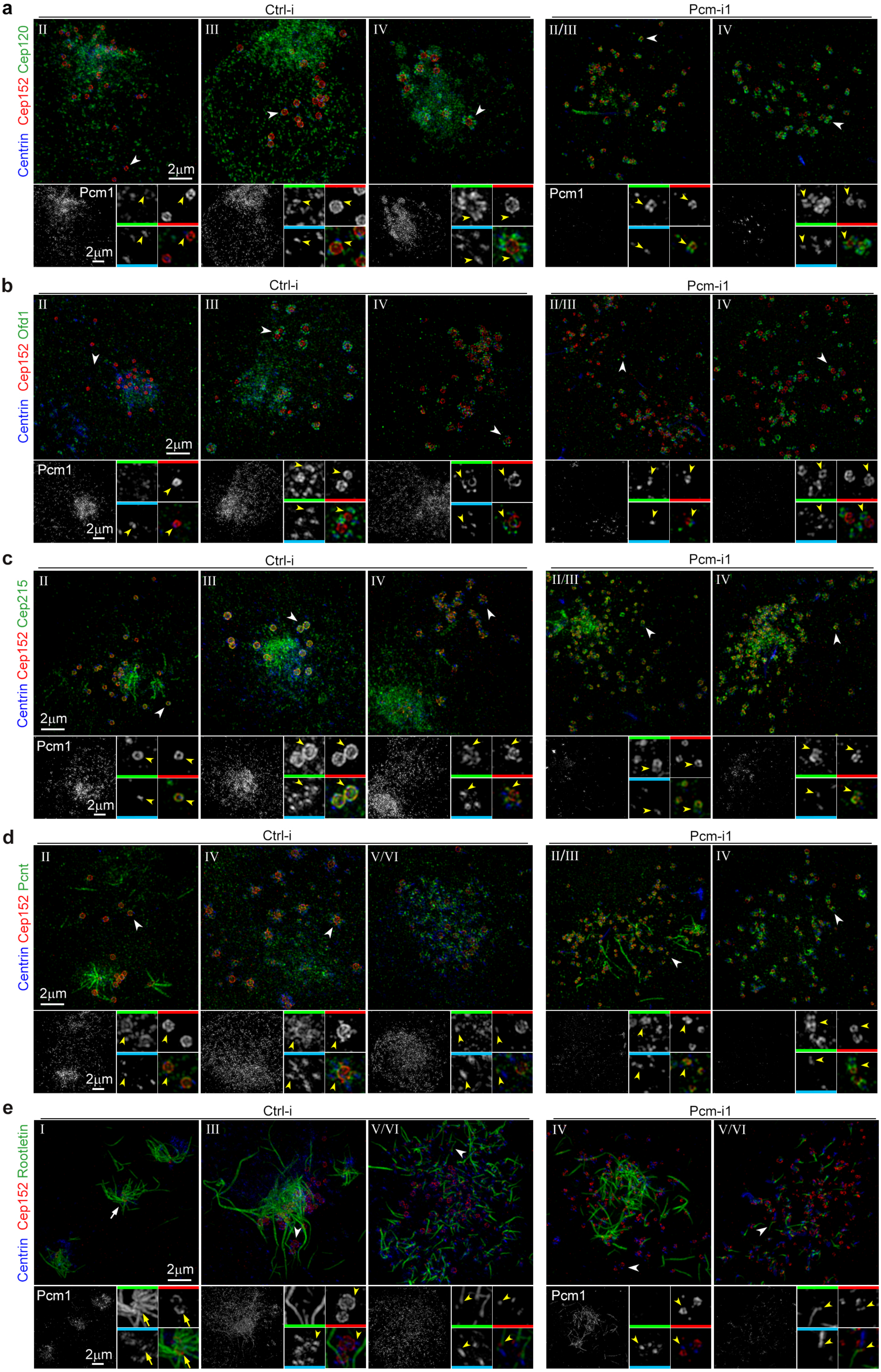

Supplementary Figure 5. Effects of Pcm1 depletion on subcellular localizations of FGM proteins (green) in mEPCs (related to Fig. 6).

mEPCs were treated and harvested as in Figure 2a. Shown are representative 3D-SIM images for Cep120 **(a)**, Ofd1 **(b)**, Cep215 **(c)**, Pcnt **(d)**, and Rootletin **(e)**. The regions indicated by white arrowheads (for deuterosomes or nascent centrioles) or an arrow (parental centriole) were magnified to show details. Yellow arrowheads or arrows point to immunofluorescent signals related to typical centrioles. Two alternative stages are indicated in cases when we have difficulty to clearly define the precise stage of some cells. Note that Rootletin tended to form numerous filaments regardless of Pcm1 in mEPCs **(e)**, which is in sharp contrast to its typical FGM localization in mTECs in stages II-V (Fig. 5d). Nevertheless, when the Pcm1-positive FGM cores were present in the cells, they still enriched Rootletin. The bright Rootletin filaments sometimes leaked into the Pcm1 channel.

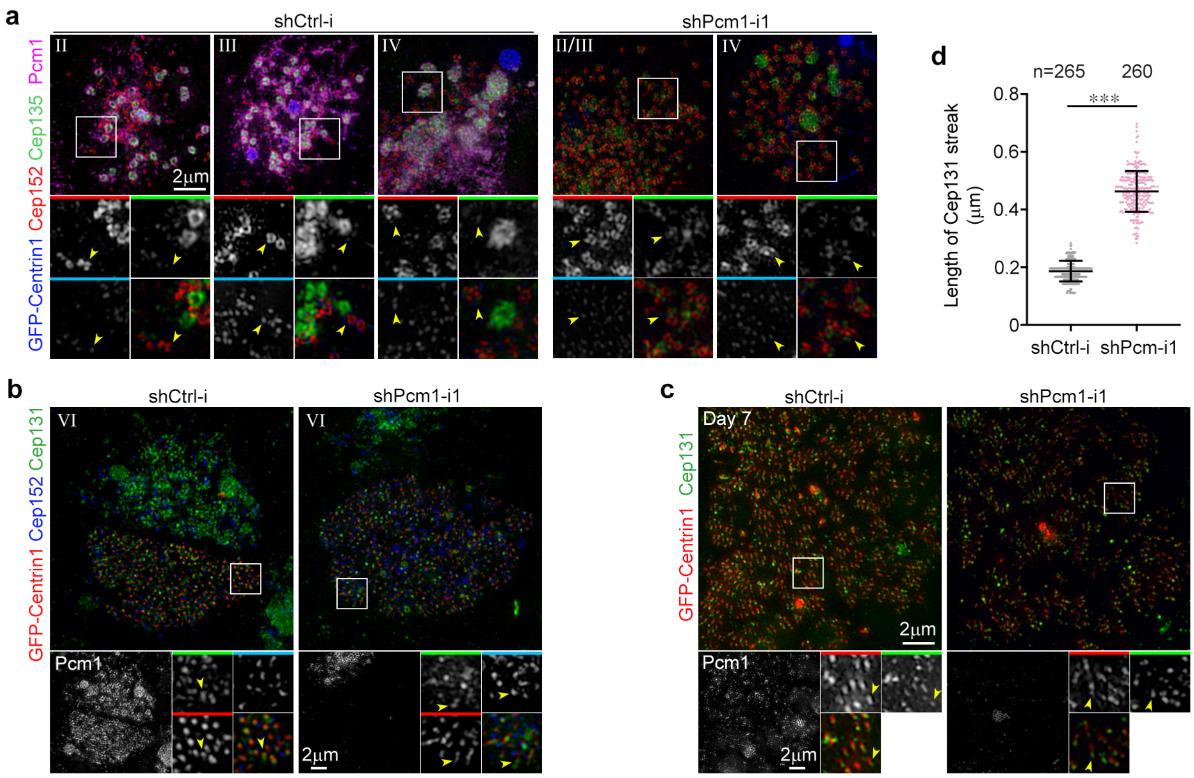

Supplementary Figure 6. Depletion of Pcm1 causes deregulated centriolar and ciliary targeting of FGM proteins in mTECs.

mTECs infected with adenovirus to express shRNAs as in Supplementary Figure 3a were fixed at day 3 **(a,b)** or day 7 **(c)** and subjected to 3D-SIM. The framed regions in micrographs **(a-c)** were magnified to show details. Arrowheads in the color-coded insets point to immunofluorescent signals related to typical centrioles. **(a,b)** Representative 3D-SIM images for Cep135 **(a)** and Cep131**(b)**. **(c,d)** Depletion of Pcm1 in mTECs increased the length of the Cep131-positive streaks. The quantification results (mean ± s.d. and sample dots) **(d)** were from 3D-SIM images **(c)** collected in two independent experiments. To minimize the orientation problem of multicilia (refer to the legends for Fig. 6e,f), only five longest Cep131 streaks were measured in each MCC. Unpaired two-tailed student's *t* test: *** P<0.001. Source data are provided in the Source data file.

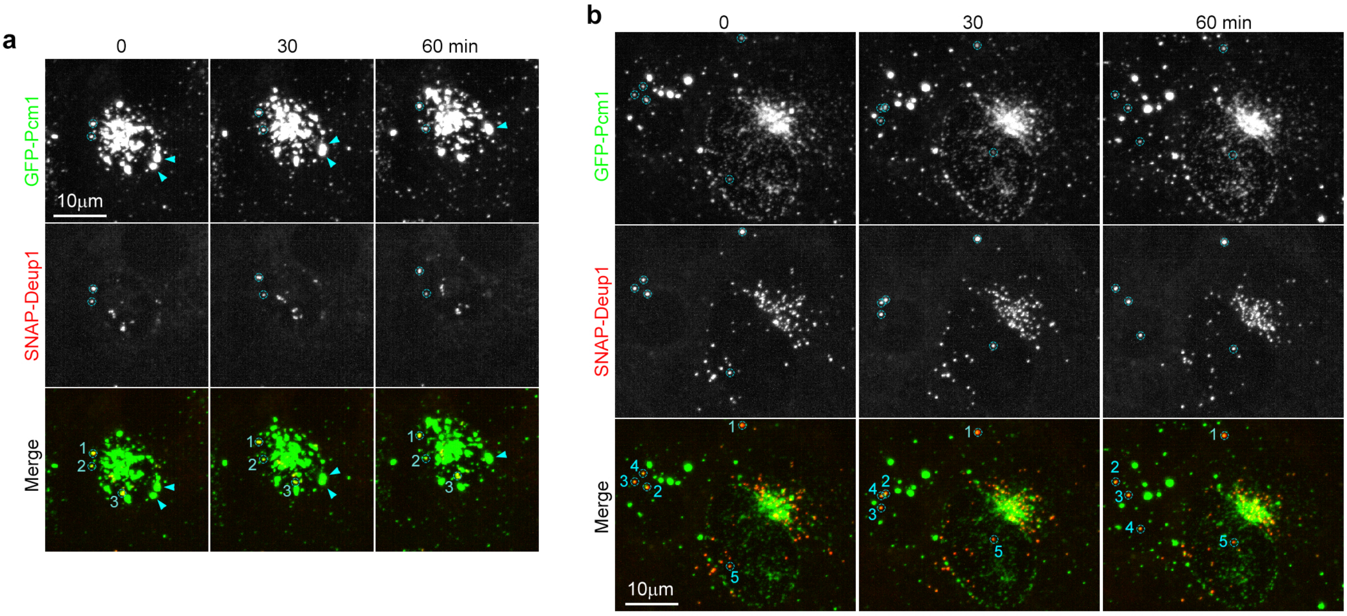

Supplementary Figure 7. FGM condensates display liquid properties and associated with deuterosomes in live mEPCs (related to Fig. 7).

(a,b) Representative frames showing fusion of FGM foci and co-movement of typical FGM foci with their associated deuterosomes. mEPCs treated to express GFP-Pcm1 and SNAP-Deup1 as in Figure 7a were live imaged at 5-min intervals. Typical deuterosomes and their associated FGMs are encircled and numbered. Arrowheads in **(a)** indicate fusion of two FGM foci. Note that most of the deuterosomes that are not numbered were also associated with FGM foci and some co-migrated with their associated FGM foci on the nuclear surface (e.g., #5 in b). Images were collected from one independent repeat.
