# Supplementary material for: Fibrogranular materials function as organizers to ensure the fidelity of multiciliary assembly
Source: Nat Commun. 2021 Feb 24;12:1273. doi: 10.1038/s41467-021-21506-8 (PMC7904937; doi:10.1038/s41467-021-21506-8)
Supplement: Supplementary file 12 — Description of additional supplementary files [file 41467_2021_21506_MOESM12_ESM.pdf]

Description of Additional Supplementary Files

Supplementary Data 1. List of candidate FGM proteins and their potential subcellular localizations.

Supplementary Data 2. List of primers used.

Supplementary Data 3. List of antibodies used.

Supplementary Movie 1. 3D reconstruction of serial z-stack EM images of an mTEC (related to Fig. 1g,h). mTECs cultured for 3 days at the air-liquid interface (ALI) were fixed and subjected to focused ion beam scanning electron microscopy (FIB-SEM). Representative x-y and x-z views are presented in Figure 1g. Note that the black triangle to the left was the leftover of the platinum layer after beaming.

Supplementary Movie 2. 3D model of an FGM core segmented from serial z-stack FIB-SEM images (related to Fig. 1g,h). Segmentation and surface generation for one of the FGM cores are demonstrated, in which the high electron-dense FGs were rendered blue and their surrounding less electron-dense materials red. Front and side views of the 3D model are presented in Figure 1h.

Supplementary Movie 3. Depletion of Pcm1 in mEPCs results in abnormal multicilia motility (related to Fig. 3e-g). mEPCs were treated with siRNA (Ctrl-i, Pcm-i1, or Pcm-i2) and cultured to day 10 as depicted in Figure 3a. Multicilia (arrowheads) were live imaged at 7-ms intervals for 1.4 sec. Image sequences in the first 343 ms are played back at 10 frames per sec (fps) for a typical field. The trajectories of four trackable cilia in the first 77 ms are shown for one of the MCCs in each sample. Scale bar, 5 μm.

Supplementary Movie 4. Depletion of Pcm1 in mTECs results in abnormal multicilia motility (related to Supplementary Fig. 3f,g). mTECs were infected with adenovirus to co-express an shRNA (shCtrl-i or shiPcm-i1) with GFP-Centrin1 and cultured to day 7 as depicted in Supplementary Figure 3a. Multicilia, stained with SiR-tubulin, were live imaged at 15-ms intervals for 3 sec. Image sequences in the first 735 ms are played back at 10 fps for

a typical field. Infected MCCs are indicated by their GFP-Centrin1-positive basal bodies (arrowheads in the 1st frame). Scale bar, 5 μm.

Supplementary Movie 5. Expression of GFP-Pcm1 rescues multicilia motility of Pcm1-depleted mEPCs (related to Fig. 3h-j). mEPCs were treated with both Pcm-i1 to deplete Pcm1 and adenovirus to express GFP-Centrin1 or an RNAi-insensitive GFP-Pcm1 as depicted in Figure 3a. At day 10, multicilia were live imaged and presented as described in the legend for Supplementary Movie 3. MCCs positive for Centrin1-GFP or GFP-Pcm1 are denoted by arrowheads in the 1st frame. Scale bar, 5 μm.

Supplementary Movie 6. Spatial relationships between FGM condensates (green) and deuterosomes (red) in mEPCs (related to Fig. 7b). 3D reconstruction was performed using z-stack images for the last frame in Figure 7b. This also verifies that the FGM condensates indicated in Figure 7b indeed fused into one.
