# Supplementary material for: Fibrogranular materials function as organizers to ensure the fidelity of multiciliary assembly
Source: Nat Commun. 2021 Feb 24;12:1273. doi: 10.1038/s41467-021-21506-8 (PMC7904937; doi:10.1038/s41467-021-21506-8)
Supplement: Supplementary file 13 — Reporting Summary [file 41467_2021_21506_MOESM13_ESM.pdf]

nature research

Corresponding author(s): Xueliang Zhu

Last updated by author(s): Jan 14, 2021

Reporting Summary

Nature Research wishes to improve the reproducibility of the work that we publish. This form provides structure for consistency and transparency in reporting. For further information on Nature Research policies, see Authors & Referees and the Editorial Policy Checklist.

Statistics

For all statistical analyses, confirm that the following items are present in the figure legend, table legend, main text, or Methods section.

n/a	Confirmed	
☐	☒	The exact sample size (n) for each experimental group/condition, given as a discrete number and unit of measurement
☐	☒	A statement on whether measurements were taken from distinct samples or whether the same sample was measured repeatedly
☐	☒	The statistical test(s) used AND whether they are one- or two-sided<br>*Only common tests should be described solely by name; describe more complex techniques in the Methods section.*
☐	☒	A description of all covariates tested
☐	☒	A description of any assumptions or corrections, such as tests of normality and adjustment for multiple comparisons
☐	☒	A full description of the statistical parameters including central tendency (e.g. means) or other basic estimates (e.g. regression coefficient) AND variation (e.g. standard deviation) or associated estimates of uncertainty (e.g. confidence intervals)
☐	☒	For null hypothesis testing, the test statistic (e.g. F, t, r) with confidence intervals, effect sizes, degrees of freedom and P value noted<br>*Give P values as exact values whenever suitable.*
☒	☐	For Bayesian analysis, information on the choice of priors and Markov chain Monte Carlo settings
☒	☐	For hierarchical and complex designs, identification of the appropriate level for tests and full reporting of outcomes
☒	☐	Estimates of effect sizes (e.g. Cohen's d, Pearson's r), indicating how they were calculated

Our web collection on statistics for biologists contains articles on many of the points above.

Software and code

Policy information about availability of computer code

Data collection	Images were collected using GE DeltaVision OMX imaging system, Leica Microsystems, Olympus SpinSR10 microscope, Olympus IX71 Microscope, FEI Helios NanoLab G3 UC FIB-SEM, and FEI Tecnai G2 Spirit TEM.
Data analysis	Data were analyzed using Image J 1.53e, Bitplane Imaris 7.6.5, Thermo Fisher Amira 6.0, Graphpad Prism 8.0.2 and ImagePro Plus 6.0.

For manuscripts utilizing custom algorithms or software that are central to the research but not yet described in published literature, software must be made available to editors/reviewers. We strongly encourage code deposition in a community repository (e.g. GitHub). See the Nature Research guidelines for submitting code & software for further information.

Data

Policy information about availability of data

All manuscripts must include a data availability statement. This statement should provide the following information, where applicable:

- Accession codes, unique identifiers, or web links for publicly available datasets
- A list of figures that have associated raw data
- A description of any restrictions on data availability

Source data are provided with this paper. Any remaining data that support the results of this study are available from the corresponding author upon reasonable request.

October 2018

Field-specific reporting

Please select the one below that is the best fit for your research. If you are not sure, read the appropriate sections before making your selection.

☒ Life sciences ☐ Behavioural & social sciences ☐ Ecological, evolutionary & environmental sciences

For a reference copy of the document with all sections, see nature.com/documents/nr-reporting-summary-flat.pdf

Life sciences study design

All studies must disclose on these points even when the disclosure is negative.

Sample size	Sample size was not predetermined with any statistical method and it is chosen dependent on availability and general guideline to achieve at least two independent experiments.
Data exclusions	No data were excluded for the analyses.
Replication	Reproducibility was confirmed. The number of independent experiments is described in the figure legends.
Randomization	We examined cells from multiple randomly-chosen fields in each experiment. All cells in the chosen fields were scored.
Blinding	Since we usually co-stained Pcm1 for cells subjected to control RNAi or Pcm1 RNAi. Blinding for our experiments is not feasible because the treatment of cells was known based on the immunostaining of Pcm1.

Reporting for specific materials, systems and methods

We require information from authors about some types of materials, experimental systems and methods used in many studies. Here, indicate whether each material, system or method listed is relevant to your study. If you are not sure if a list item applies to your research, read the appropriate section before selecting a response.

Materials & experimental systems

n/a	Involved in the study	
☐	☒	Antibodies
☐	☒	Eukaryotic cell lines
☒	☐	Palaeontology
☐	☒	Animals and other organisms
☒	☐	Human research participants
☒	☐	Clinical data

Methods

n/a	Involved in the study	
☒	☐	ChIP-seq
☒	☐	Flow cytometry
☒	☐	MRI-based neuroimaging

Antibodies

Antibodies used	All antibodies used in this work were listed in supplementary table 2 with source information, catalog number and dilution information.
Validation	All antibodies commercially available have been validated by the manufacturer. The validation of Rabbit anti-Cep131, Rabbit anti-Cep135, Rabbit anti-Cep120, Rabbit anti-Rootletin, Rabbit anti-Pcnt, Rabbit anti-Cep215, Rabbit anti-Cep76, Rabbit anti-Cep290, Rabbit anti-Ofd1 and Rat anti-Pcm1 based on immunofluorescence staining and immunoblotting of mouse tracheal epithelial cells and mouse ependymal cells. Rabbit anti-Ofd1, Rabbit anti-Deup1, Rabbit anti-Cep164, Rabbit anti-Rsph4a, Chicken anti-Cep152, Guinea pig anti-Cep162 and Guinea pig anti-Hydin was validated in previously published papers.

Eukaryotic cell lines

Policy information about cell lines

Cell line source(s)	HEK293T (ATCC), HEK293A (Thermo Fisher)
Authentication	The cell lines have been authenticated by the vendor. No further authentication was performed.
Mycoplasma contamination	Mycoplasma contamination was tested and found negative.
Commonly misidentified lines (See ICLAC register)	No commonly misidentified cell lines were used.

Animals and other organisms

Policy information about studies involving animals; ARRIVE guidelines recommended for reporting animal research

Laboratory animals	Wild-type P0 and 4-week male or female C57BL/6J mice were used for primary cell culture. The mice were housed under specific-pathogen-free (SPF) conditions in cages and a 12/12-hr light/dark photoperiod at 20-26 °C. The humidity of the housing room was maintained at 40-70% humidity.
Wild animals	No wild animals were used.
Field-collected samples	No field-collected samples were used.
Ethics oversight	Experiments involving mouse tissues were performed in accordance with protocols approved by the Institutional Animal Care and Use Committee of CAS Center for Excellence in Molecular Cell Science, Institute of Biochemistry and Cell Biology, Chinese academy of Sciences.

Note that full information on the approval of the study protocol must also be provided in the manuscript.
